# Editorial: Immersive Media in Connected Health

**DOI:** 10.3389/fdgth.2021.697336

**Published:** 2021-09-08

**Authors:** Panagiotis E. Antoniou, Daphne Economou, Alkinoos Athanasiou, George Tsoulfas

**Affiliations:** ^1^Laboratory of Medical Physics and Digital Innovations, Department of Medicine, School of Health Sciences, Aristotle University of Thessaloniki, Thessaloniki, Greece; ^2^Department of Computer Science and Engineering, University of Westminster, London, United Kingdom; ^3^First General Surgery Department, Papageorgiou General Hospital/Aristotle University of Thessaloniki, Thessaloniki, Greece

**Keywords:** mixed reality, medical education, surgery, telepresence, 3D printing

## Introduction

According to Fortune Business Insights, the market size of extended reality (XR), a blanket term comprising augmented, virtual, and augmented reality (AR/VR/MR), is estimated to grow to 30.4 billion USD by 2027 from an estimated 1.56 billion USD (1). This simple figure outlines more succinctly than any literary review the explosive growth of XR in the healthcare sector. With XR being only one vista in the whole landscape of immersive media in connected health, the scope of this research study becomes readily apparent. Medical image fusion, educational immersive visualizations, web-based virtual scenarios, and XR interventions for mental and physical health are all parts of the research ecosystem of this topic.

This special issue aims at contemporary immersive media research study as it pertains to healthcare. From the whole spectrum of XR to a diverse landscape of image processing and fusion, educational simulations, and technical/usability issues in proliferating immersive media in the whole connected healthcare ecosystem, this special issue aims to explore both the technologies of immersive media and their impact on contemporary healthcare.

On the educational front, virtual patients (VPs), with current web-based rapid development and deployment cycles, are ubiquitously present in the healthcare curricula. Meaningfully combining immersive three-dimensional (3D) virtual environments with VPs leads medical training to new aspects of open social learning. Learners can absorb educational content at their own pace and engage in an experiential way and not only feel the presence but also affect the sense of presence in the training event (2). Developing such resources and repurposing them for virtual reality (VR) are a rather straightforward task, that is, one, however, that must adhere to sound game-informed design principles and narrative techniques to achieve measurable impact both in learning outcomes and engagement (3).

However, education is not the only healthcare field that XR applies. Preoperative surgical planning using mixed reality (4) and 3D prints for patient-specific surgical approaches are proliferating (5). Haptic controls in conjunction with XR visualization and novel medical image co-registration and fusion are becoming common practices in surgical training and preoperative preparation.

Beyond “hard” medicine applications, the whole spectrum of immersive health is benefited through healthcare interventions. The general public and targeted sensitive groups of people are benefiting from physical and mental wellness interventions based on immersive media such as XR, m-health, or even virtual environment ecosystems (6).

## Outline of Contributions

In this context, our topic attracted a diverse collection of studies.

Starting from the core need for surgical guidance, Drakopoulos et al. combined non-rigid registration of preoperative operative data with mixed reality presentation in order to provide an affordable, fast, and immersive solution that is able to tackle the intraoperative brain deformation issue in brain tumor resections. During resection, especially of significant tumor volumes, navigation systems based on preoperative imaging lose significant portions of accuracy and guidance value due to dynamic brain shifts. The authors used an Adaptive Physics-Based Non-Rigid Registration method (A-PBNRR) and multi-tissue mesh adaptation to register preoperative and intraoperative MRI on glioma surgery data of real patients and evaluated registration accuracy using visual assessment, Hausdorff distance-based metric, and a landmark-based approach using anatomical points identified by a neurosurgeon. Their approach reported several positive outcomes: It significantly improved the accuracy of deformable registration compared to traditionally employed methods; it was sufficiently fast for intraoperative application; and when coupled with Mixed Reality (MR), it offers an affordable alternative solution to current neuronavigation systems.

Moving to core medical education, the study by Jivram et al. explored the use of Second Life virtual 3D environment as a more realistic space for deploying virtual scenarios. Collaborative learning through case-based or problem-based learning (PBL) is an established way to cultivate workplace knowledge associated with specific competencies. At the St George's University of London, an interactive online form of decision-based PBL (D-PBL) was developed using web-based VPs, which was subsequently transferred to the Second Life virtual environment. It is interesting to note that while a small number of students found the Second Life experience more engaging in terms of realism, most students favored a simpler more concise interaction of the web-based VPs. In that context, providing the learner with the essential topical knowledge in a simple engaging medium trumped the increased immersion of the virtual environment.

Deepening the exploration of educational themes, Pieterse et al. researched how augmented reality provides us with the necessary technology in order to enhance the experience of the real world with computer-generated information and a variety of auditory, haptic, and visual stimuli. This opens a whole new world of educational opportunities (among many others), which have the potential to transform education at all different levels. In this study, the authors developed an augmented reality application for the presentation of shortness of breath (dyspnea). This was subsequently successfully implemented in the medical curriculum, where its value was confirmed by medical students. The authors provide a detailed description of an elegant and elaborate process, which has the potential to provide us with potent, novel educational tools.

Finally concluding the topic, an important technical usability hurdle is tackled by Schmidt et al. The issues of the unpleasant side effects caused by VR headset to users such as cybersickness and sensory disorders have been well studied. However, there has been almost no exploration of these problems with individuals with autism or other cognitive disabilities. The study with the title “process-model for minimizing adverse effects when using head mounted display-based virtual reality for individuals with autism” is an initial attempt to address this gap by conducting a study in two separate, independent research sites—one in the United States and one in the United Kingdom. The results of the study assert that the proposed guidelines could provide clarity in terms of design and implementation of headset-based VR for individuals with autism spectrum disorders, guide implementations of future researchers and practitioners, and contribute to minimizing and controlling for potential adverse effects.

## Concluding Remarks

While the field is quite extensive, it is auspicious that the scope of the collected studies has been diverse enough to traverse most of its admittedly wide scope. It is within this auspicious context that we invite the reader to traverse the nuances of an eclectic collection of research studies on this topic.

## Author Contributions

PA: general section authorship and review of contributions to the Research Topic. DE, AA, and GT: review of contributions to the Research Topic. All authors contributed to the article and approved the submitted version.

## Conflict of Interest

The authors declare that the research was conducted in the absence of any commercial or financial relationships that could be construed as a potential conflict of interest.

## Publisher's Note

All claims expressed in this article are solely those of the authors and do not necessarily represent those of their affiliated organizations, or those of the publisher, the editors and the reviewers. Any product that may be evaluated in this article, or claim that may be made by its manufacturer, is not guaranteed or endorsed by the publisher.
